# Annotations

**Published:** 1909-06-12

**Authors:** 


### ANNOTATIONS.

### Sunday Rest for Medical Men.

THE following free translation of a notice which the members of L'Union Médicale de Huy have caused to be printed and distributed to their patients is instructive, as showing the trend of the times towards trades-unionism, even in things medical. Professional co-operation in this district of France has resulted in the announcement that, "Since Sunday rest is as indispensable to the medical profession as to any other trade or profession, but since it would be difficult to organise to this end a rotation corresponding to that established by the pharmacists, the Union Médicale thinks it proper to call the attention of the public, in a practical manner, to the necessity of this rest for the faculty. To this effect the society decides, in conformity with the action which has already been taken in several other districts in this country, to put a check on the visits and consultations, often in no way urgent, which are demanded by the public of medical practitioners at all hours on Sundays. It has been decided that henceforward every visit asked for by patients on Sundays and public holidays after 9 A.M.—that is to say, after the medical practitioner's usual hour for commencing his morning round, shall be considered as a visit of urgency, and shall consequently be charged for at double the ordinary rates." The general practitioners of these islands will no doubt agree with us that this decision is neither unreasonable nor impracticable.

### The Council's Report of the British Medical Association.

IN readiness for the forthcoming seventy-seventh annual meeting of the British Medical Association in July next at Belfast, the Council has now issued its Annual Report, which appeared in the supplement to the issue of the *British Medical Journal* of May 22. Several topics of much interest, relating to the Association's work during the past twelve months, are dealt with by the Council, and a good general survey of the year's progress and activity may be gained from a study of the Report; but we have only space to deal here with two or three points. The completion of the work of rebuilding the Association's central building and Journal offices, off the Strand, naturally forms the subject of general satisfaction. The Council may, indeed, congratulate itself and the Association upon the imposing structure which now provides a home, worthy in every respect of the influence and numerical strength of this immense professional corporation. Now that the absurd and highly artificial controversy, which raged last summer around the sculptural decoration of this building, has long gone the way of all such journalistic sensations, the Council may reasonably feel that these new quarters form a definite addition to the dignity, comfort, and sphere of utility of the British Medical Association. A source of regret and concern to the Council is provided by the current statistics of membership. Whatever may be the causes, the fact that the net increase of membership has fallen from 1,266 in 1906, through 131 in 1907, to 39 in 1908, must provoke anxious consideration on the part of the authorities. Analysis of membership figures shows that resignations in the past three years numbered respectively 485, 1,050 and 584, which will perhaps be regarded by the current executive with mixed feelings. New members in 1906 numbered 2,171; in 1907, 1,527; and in 1908, 1,023; and this very roughly corresponds to the decline in additions to the Medical Register during the same periods; but nevertheless there is some force in the regret which the Council expresses that, in view of all that the Association is endeavouring to accomplish on behalf of the medical profession, the increase in membership has not been more marked. The financial statement concludes thus: "The Association, like any other public body, must stand or fall by the soundness of its financial position. If demands upon the resources of the Association continue to grow in the same degree, the most important question members will have to decide is, not so much what the Association is going to do, but how it is going to do it." Here, indeed, is a matter which may well occupy the spare thoughts of those members to whom their Association is something more than a mere professional trades union, which supplies them with a weekly periodical.

### Research in Nervous Disease.

WHETHER from the point of view of pure reason the non-application of hospital funds to purposes of research is justifiable or not, is not now a question of practical politics. Public opinion holds that charitable funds must not be applied to this particular method of advancing the common weal; though public opinion is ever labile, and its education is advancing, yet meanwhile hospitals must pay heed to its views on this matter. In 1904 a Nervous Diseases Research Fund was established, and was commended to the public by Mr. Chamberlain, by the President of the Royal Society, and by others. This fund was to enable some use to be made of the enormous mass of clinical material and records of the National Hospital for the Paralysed and Epileptic, Queen Square, and hitherto it has proved sufficient to meet the expenses of the researches entered upon. The time has, however, come when, according to the committee under whose supervision the money is expended, it is necessary to make a wider appeal for support. This appeal, to which we are very glad to afford publicity, is signed by names which are household words to every student of neurology; and members of the medical profession can forward the cause by assuring any who may ask their advice on this subject of the unequalled standing of these neurologists. It is not at all rare to hear students and recently qualified men excuse themselves for an admitted ignorance of nervous diseases by a remark that since all are incurable there is little object in learning to differentiate one from another. Even were it true, as it fortunately is not, that all or nearly all nervous diseases are incurable, the argument in favour of special research into such a subdivision of medicine would be strengthened rather than the reverse.

